# Viperin Regulates Cellular Lipid Metabolism during Human Cytomegalovirus Infection

**DOI:** 10.1371/journal.ppat.1003497

**Published:** 2013-08-01

**Authors:** Jun-Young Seo, Peter Cresswell

**Affiliations:** 1 Department of Immunobiology, Howard Hughes Medical Institute, Yale University School of Medicine, New Haven, Connecticut, United States of America; 2 Severance Biomedical Science Institute, Yonsei University College of Medicine, Seoul, Korea; Oregon Health and Science University, United States of America

## Abstract

Human cytomegalovirus (HCMV) has been shown to induce increased lipogenesis in infected cells, and this is believed to be required for proper virion envelopment. We show here that this increase is a consequence of the virus-induced redistribution of the host protein viperin to mitochondria and its capacity to interact with and block the function of the mitochondrial trifunctional protein (TFP), the enzyme that mediates fatty acid-β-oxidation. The resulting decrease in cellular ATP levels activates the enzyme AMP-activated protein kinase (AMPK), which induces expression of the glucose transporter GLUT4, resulting in increased glucose import and translocation to the nucleus of the glucose-regulated transcription factor ChREBP. This induces increased transcription of genes encoding lipogenic enzymes, increased lipid synthesis and lipid droplet accumulation, and generation of the viral envelope. Viperin-dependent lipogenesis is required for optimal production of infectious virus. We show that all of these metabolic outcomes can be replicated by direct targeting of viperin to mitochondria in the absence of HCMV infection, and that the motif responsible for Fe-S cluster binding by viperin is essential. The data indicate that viperin is the major effector underlying the ability of HCMV to regulate cellular lipid metabolism.

## Author Summary

Virus infection induces the production of interferons, which in turn stimulate the production of a set of proteins that often have antiviral functions. One of these interferon-inducible proteins is viperin, the product of the human *Rsad2* gene. Human cytomegalovirus (HCMV) paradoxically induces expression of viperin independently of the interferon response, and we previously showed that a virus-encoded protein transports the induced viperin to mitochondria where it interferes with fatty acid b-oxidation, a major energy generating system of the cell. We show here that this ultimately results in enhanced lipid synthesis by the infected cell that is essential for production of infectious virus. The mechanism involves sensing the depletion in ATP levels caused by inhibition of fatty acid b-oxidation by the enzyme AMP-induced protein kinase. This induces a cascade of events that result in the increased transcription of genes encoding lipogenic enzymes and consequent lipid biogenesis that is needed by the virus for adequate membrane envelope formation. Thus HCMV uses the interferon-inducible protein viperin, known to be antiviral for other viruses, even for HCMV itself if viperin is pre-expressed in cells prior to infection, to modulate the metabolic status of the cell to facilitate its replication.

## Introduction

Human cytomegalovirus (HCMV) is associated with acute and chronic disease in both healthy and immunocompromised populations [1], [2], [3]. A characteristic of HCMV is that it modulates the metabolism of an infected cell in ways that favor viral replication [4], [5], [6]. HCMV infection has been shown to induce the expression of glucose transporter 4 (GLUT4) and its translocation to the cell surface, which results in an increase in cytoplasmic glucose that is used for *de novo* fatty acid biosynthesis [4], [5], [6], [7], [8]. The increase in GLUT4 expression during HCMV infection has been shown to result from activation of AMP-activated protein kinase (AMPK) [9]. The increase in fatty acid biosynthesis leads to the accumulation of lipids that are used for formation of the viral envelope [10], [11]. Consistent with this, pharmacological inhibition or siRNA-mediated knockdown of fatty acid synthetic enzymes reduces HCMV replication [5], [10].

Two major classes of transcription factors regulate *de novo* fatty acid synthesis by inducing the expression of lipogenic enzymes. These are sterol regulatory element binding proteins (SREBPs) and carbohydrate responsive element binding protein (ChREBP), which are insulin- and glucose-responsive transcription factors, respectively [12], [13], [14], [15], [16], [17]. In cells with sufficient sterol levels, SREBPs remain in the endoplasmic reticulum (ER). When sterol levels are depleted, the insulin-stimulated SREBPs are transported to the Golgi where they are cleaved to a mature form and translocated into the nucleus. The cleaved forms of SREBPs up-regulate the expression of lipogenic genes [13], [14], [15]. ChREBP is also an important transcriptional regulator for *de novo* lipogenesis. Glucose activates ChREBP by regulating its redistribution from the cytosol to the nucleus by a phosphorylation dependent mechanism [18], [19]. Recently, it was shown that in adipose tissue GLUT4-mediated glucose uptake induces ChREBP, which activates *de novo* lipogenesis [20]. HCMV infection has been shown to induce the cleavage of SREBPs and also to maintain constitutive lipid synthesis by overriding sterol feedback control during infection [10], [11]. However, the fundamental mechanisms responsible for HCMV-induced activation of lipid synthesis remain poorly understood.

Upon infection HCMV directly induces the interferon (IFN)-inducible iron-sulfur (Fe-S) cluster-binding protein, viperin [21], [22], [23], and we recently showed that the HCMV-encoded vMIA protein binds viperin and translocates it to mitochondria where it inhibits fatty acid β-oxidation [24]. This results in reduced cellular ATP levels and disruption of the actin cytoskeleton, previously shown to increase viral infectivity [25], [26], [27], [28]. Here we demonstrate that the induced viperin is also responsible for the increases in AMPK activity, GLUT4 and lipogenic enzyme transcription, and enhanced lipid synthesis observed in HCMV-infected cells. The interaction of viperin, but not a mutant lacking the Fe-S cluster binding motif, with the mitochondrial trifunctional protein (TFP) that mediates fatty acid β-oxidation [29], [30] is critical for these effects. These data suggest that viperin is the key molecule that regulates lipid metabolism during HCMV infection.

## Results

### HCMV infection-induced viperin mediates multiple metabolic effects, culminating in enhanced lipogenesis

**Figure 1:**
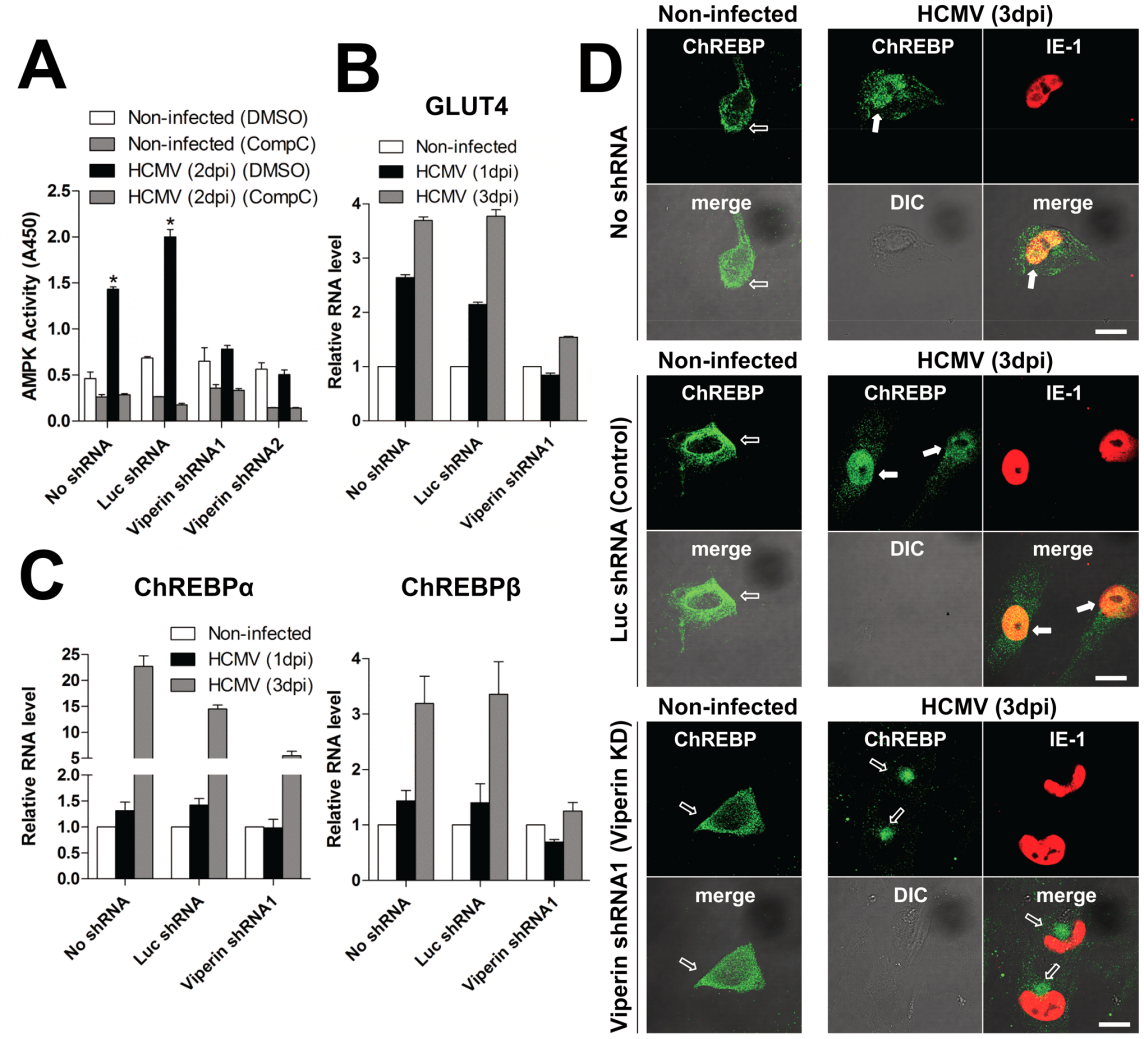
Viperin expression is necessary for HCMV-induced metabolic effects. HFtelo cells stably expressing no shRNA, control luciferase (Luc) shRNA or viperin shRNAs were infected with HCMV at an moi of 2 for the indicated days. (**A**) AMPK activity. Cells were treated with DMSO or the AMPK inhibitor, Compound C (CompC; 5 µM). At 2 days post infection (dpi), the cells were harvested in lysis buffer and the lysates from 2.5×10^4^ cells were assayed for AMPK activity. Data are presented as mean ± SEM of duplicate samples and are representative of two individual experiments. *, *P*<0.01. (**B** and **C**) mRNA levels of GLUT4 (**B**), ChREBPα and ChREBPβ (**C**). Total RNA was isolated, and the mRNA levels were measured by quantitative RT-PCR and normalized to β actin mRNA. Data are presented as means ± SEM of triplicate samples and are representative of three individual experiments. (**D**) Cells were stained with antibody specific to ChREBP and antibody to the HCMV protein IE-1 to identify infected cells. The filled arrows indicate ChREBP localized to the nuclei, and the open arrows indicate ChREBP localized to cytoplasm. Fifty infected cells were examined for ChREBP nuclear localization. ChREBP was detected at the nuclei in over 50% of HCMV-infected control cells and less than 1% of viperin knockdown cells. Scale bar, 20 µm.

Viperin interaction with the mitochondrial trifunctional protein (TFP) depletes cytoplasmic ATP [24]. ATP depletion generally results in AMP accumulation, which leads to the activation of AMPK [31], [32], [33], and AMPK has been shown to be important for HCMV-mediated alterations in metabolism [9]. To test whether viperin expression mediated by HCMV infection is required for AMPK activation, immortalized human fibroblasts (HFtelo), expressing either no shRNA (wild type), luciferase-specific (Luc) shRNA (control), or two different viperin shRNAs (viperin knockdown), were infected as previously described (Figure S1A) [24]. The AMPK activity in control cells at 2 days post infection (dpi) was increased by 3-fold over that in non-infected cells, while no change was observed in cells expressing viperin-specific shRNAs (Figure 1A). The addition of Compound C, a specific AMPK inhibitor [34], suppressed the measured enhancement.

Activation of AMPK induces GLUT4 expression [9], [35], and HCMV infection has been shown to activate GLUT4 independently of its normal control mechanisms, which include external glucose concentration, ATP-citrate lyase (ACL) activation, insulin stimulation, and Akt activity [8]. We asked whether viperin expression affects GLUT4 expression and GLUT4-mediated glucose uptake. HFTelo cells expressing no shRNA, control shRNA or viperin shRNAs were infected with HCMV and GLUT4 mRNA was measured by quantitative RT-PCR (Figure 1B). The mRNA levels significantly increased in infected cells expressing no shRNA or Luc shRNA, while no increase occurred in the viperin knockdown cells at 1 dpi and increases at 3 dpi were minimal (Figure 1B). These data indicate that viperin expression is required for induction of GLUT4 during HCMV infection. Because GLUT4 expression should increase glucose import, we also measured the levels of glycolysis in the infected control and viperin-specific shRNA-expressing cells. Consistent with GLUT4-dependent glucose accumulation, glycolysis was increased in control cells but not viperin knockdown cells (Figure S1B).

ChREBP is a glucose-responsive transcription factor that activates the transcription of lipogenic enzymes and is highly regulated by GLUT4 in adipose tissue [20]. To determine if ChREBP plays a role in HCMV infection, we measured the mRNA levels of ChREBPα (the canonical isoform) and ChREBPβ (a novel isoform) [20] in control and viperin knockdown cells (Figure 1C). Both transcripts of ChREBP were increased upon HCMV infection, and in both cases the increase was significantly blocked in cells expressing viperin shRNA (Figure 1C). This was particularly evident at 3 dpi. ChREBP translocates from the cytoplasm to the nucleus to function as a transcription factor, and we examined the intracellular localization of ChREBP in HCMV-infected cells. ChREBP was detected in both cytoplasm and nucleus of control cells (over 50% of HCMV-infected cells) at 3 dpi, while in the viperin knockdown cells it was not present in the nucleus (Figure 1D).

**Figure 2:**
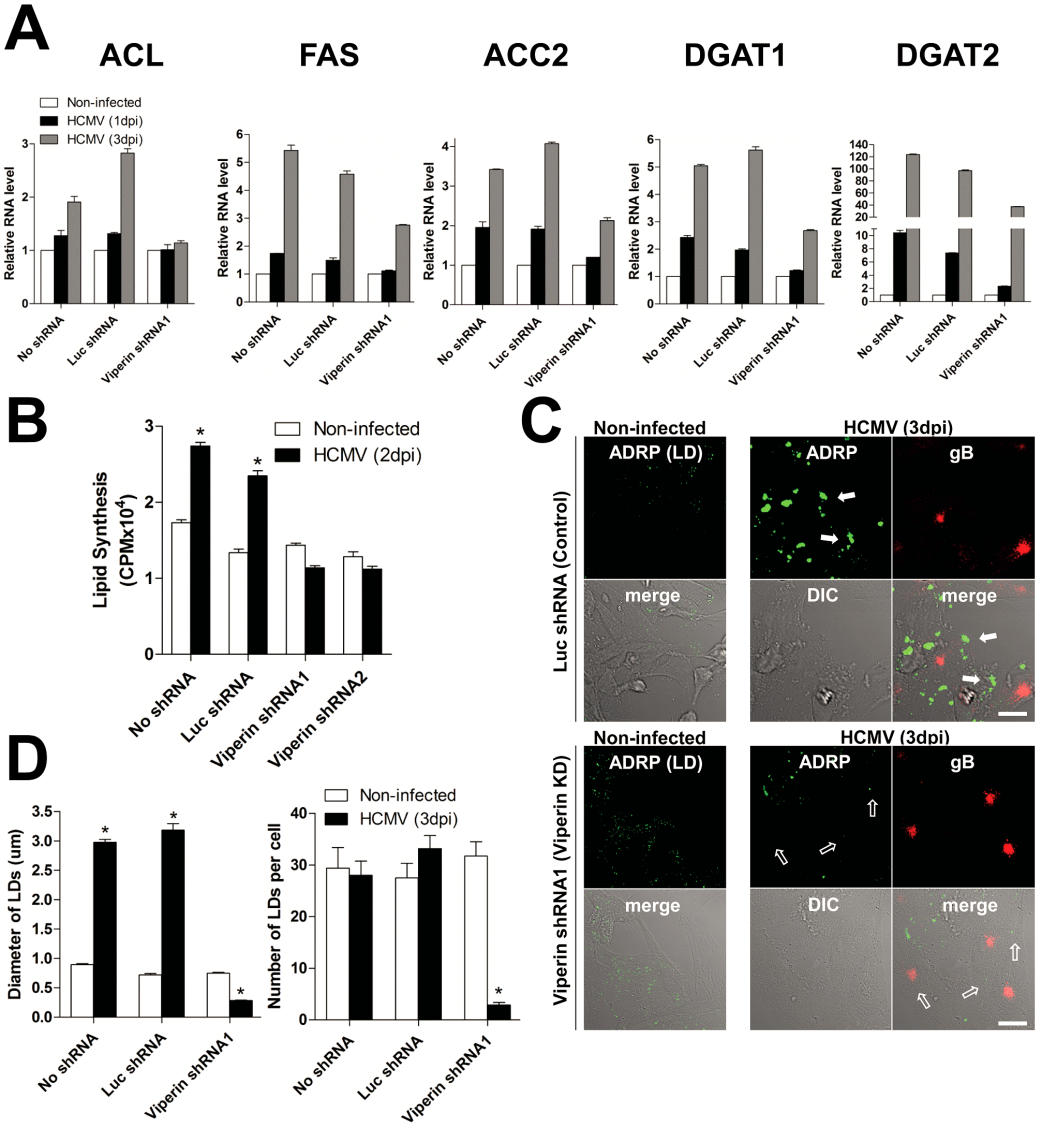
Viperin expression induces HCMV-induced lipogenesis. HFtelo cells stably expressing no shRNA, control luciferase (Luc) shRNA or viperin shRNAs were infected with HCMV at an moi of 2 for the indicated days. (**A**) Lipogenic enzyme mRNA levels. Total RNA was isolated, and the mRNA levels were measured by quantitative RT-PCR and normalized to β actin mRNA. Data are presented as means ± SEM of triplicate samples and are representative of three individual experiments. ACL, ATP-citrate lyase; ACC2, acetyl-coenzyme A (CoA) carboxylase 2; FAS, fatty acid synthase; DGAT1 and DGAT2, diacylglycerol acyltransferases 1 and 2. (**B**) Total lipid synthesis. The infected cells were labeled with [^14^C]-acetate for 3 hr. Total lipids from 1×10^5^ cells were extracted and [^14^C] incorporation assessed. Data are presented as mean ± SEM of triplicate samples and are representative of two individual experiments. *, *P*<0.0001. (**C**) Cells were stained with antibodies specific to adipose differentiation-related protein (ADRP), a marker for lipid droplets (LDs), and gB, an HCMV glycoprotein. The filled arrows indicate the accumulated LDs, and the open arrows indicate barely detectable LDs in HCMV-infected viperin knockdown cells. Scale bar, 20 µm. (**D**) Quantification of LDs. LD diameter: mean of 500 LDs ± SEM.; LD number: mean of 50 cells ± SEM *, *P*<0.0001.

ChREBP translocation to the nucleus induces lipogenic enzyme transcription, and HCMV infection is known to increase *de novo* fatty acid synthesis by inducing expression of lipogenic enzymes [10], [11]. We therefore measured mRNA levels for the key lipogenic enzymes ATP-citrate lyase (ACL), Acetyl-coenzyme A (CoA) carboxylase (ACC) 2, fatty acid synthase (FAS), diacylglycerol acyltransferase (DGAT) 1, and DGAT2. All of these transcripts were increased upon HCMV infection of cells expressing no shRNA or Luc shRNA, and in all cases the increase was substantially blocked in cells expressing viperin-specific shRNA (Figure 2A). This was particularly evident at 3 dpi.

Interestingly, SREBP1 cleavage was independent of viperin expression during HCMV infection (Figure S2A), although inhibition of SREBP1 cleavage has been shown to reduce lipid synthesis and impair HCMV growth [10], [11]. The results suggest that in HCMV-infected fibroblasts, like in adipose tissue, GLUT4-mediated glucose uptake and *de novo* lipogenesis are dominantly regulated by ChREBP rather than SREBP1, and that this process is viperin-dependent. To address this we measured lipogenesis in cells expressing GLUT4- and ChREBP-specific shRNAs during HCMV infection (Figure S2B and S2C). The mRNA levels of ChREBP and lipogenic enzymes were substantially lower in GLUT4 knockdown cells than control cells during HCMV infection (Figure S2C). Like in control cells, GLUT4 mRNA content was increased in ChREBP knockdown cells, while the increases in lipogenic enzyme mRNA levels were substantially blocked at 1 and 3 dpi (Figure S2C). The data support a mechanism in which viperin induces GLUT4 expression, increasing glucose uptake and thus ChREBP, which in turn activates *de novo* lipogenesis during HCMV infection.

The increase in lipogenic enzyme levels upon HCMV infection results in increased lipid synthesis and lipid droplet (LD) induction [10], [11]. We therefore measured total lipid synthesis in the HCMV-infected cells using ^14^C-labeled acetate. Total lipid synthesis in control cells at 2 dpi during a 3 hour labeling period was increased by 2.5-fold over that in non-infected cells, while no change was observed in the viperin shRNA-expressing cells (Figure 2B). We also examined formation of LDs, which are storage compartments for triglycerides and long chain fatty acids [36], [37]. LDs increased in diameter by approximately 3–4-fold in the HCMV-infected control cells at 3 dpi, while both the number and size of the induced LDs were dramatically reduced in viperin shRNA expressing cells (Figure 2C and D). No changes in LD number or size were observed upon viperin knockdown without HCMV infection. These data suggest that in the absence of viperin HCMV consumes pre-existing lipids, reducing the size and number of LDs, but cannot induce lipids to replenish them.

Having previously observed that HCMV-encoded vMIA is responsible for targeting viperin to the mitochondria [24], we asked whether vMIA expression affects viperin-dependent lipogenesis during HCMV infection. MRC5 fibroblasts were infected with RVHB5 (control wild type HCMV), or RVHB5ΔvMIA, a mutant of this virus lacking vMIA, in the presence of ZVAD-FMK, a broad-spectrum caspase inhibitor that prevents apoptosis otherwise induced by HCMV lacking vMIA [38]. In contrast to the results with cells infected by wild type virus, in RVHB5ΔvMIA-infected cells viperin did not substantially co-localize with mitochondria at 1 dpi (Figure S2D) and lipogenesis was substantially blocked (Figure S2E). These results indicate that viperin translocation to the mitochondria by vMIA is required for the lipogenesis induced during HCMV infection.

### Viperin-dependent lipid synthesis is required for formation of HCMV viral envelope and production of infectious virus

**Figure 3:**
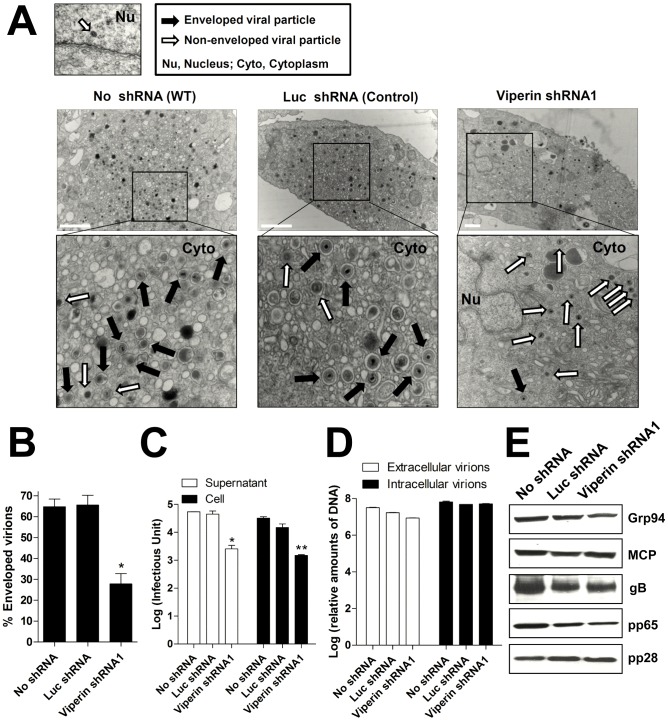
Viperin-dependent lipid synthesis is required for formation of HCMV viral envelope and production of infectious virion. (**A**) Transmission electron micrographs of HCMV-infected HFtelo cells stably expressing the indicated shRNAs. Monolayers were infected with HCMV at an moi of 2 and processed for EM at 7 dpi. Multiple frames from each sample were imaged and photographed. Particles from a representative cell are shown. White and black arrows indicate non-enveloped particles and enveloped particles, respectively. Nu, nucleus; Cyto, cytoplasm. Scale bar, 1 µm. (**B**) The numbers of enveloped and non-enveloped particles were counted in each frame and calculated as a ratio of enveloped particles to total particles in the cytoplasm of infected cells. The graphs indicate the mean percent of the enveloped particles per total 20–50 particles in each frame (from >10 cells of each sample) ± SEM *, *P*<0.0001. (**C**) HFtelo cells expressing the indicated shRNAs were infected with HCMV at an moi of 0.2, and supernatants and cells were harvested at 6 dpi. Virus yield was quantified by a fluorescence-based virus infectivity assay. Data are presented as means ± SEM of duplicate samples and are representative of two individual experiments. *, *P*<0.01; **, *P*<0.005. (**D**) Viral genome copy numbers in viral particles harvested from supernatants and cells at 6 dpi (initial moi of 2). The viral DNAs were extracted and assayed by real-time PCR. (**E**) Expression of HCMV viral proteins, MCP, gB, pp65, and pp28 in HFtelo cells expressing the indicated shRNAs at 6 dpi (initial moi of 2). Each protein was detected by immunoblot using specific monoclonal antibodies. Grp94 served as a protein-loading control.

Enhanced lipid synthesis is thought to provide the membrane necessary for proper HCMV envelope formation. To explore the role of viperin in this process we analyzed the viral particles generated in the presence and absence of viperin by quantitating cytoplasmic non-enveloped particles (capsids and tegumented capsids) and enveloped particles (double-layered particles formed by tegumentation and secondary envelopment in the secretory pathway) in control and viperin knockdown cells by electron microscopy, late in infection to allow accumulation of quantifiable virions (Figure 3A). In normal cells or those expressing the control shRNA approximately 65% of the cytoplasmic viral particles were enveloped and this was reduced to approximately 25% in the viperin shRNA expressing cells (Figure 3B). We also observed a substantial decrease in the production of infectious extracellular and intracellular virus in the cells expressing viperin shRNA (Figure 3C). However, the viral genome copy numbers present in both extracellular and intracellular particles produced from the cells after infection were similar, regardless of shRNA expression (Figure 3D). In addition, we observed no change in the expression of the intracellular HCMV viral proteins MCP (a capsid protein), gB (an envelope protein), and pp65 and pp28 (tegument proteins) (Figure 3E). The data indicate that cells with impaired viperin expression produce a similar number of viral particles but many of them are non-infectious because of defective envelopment.

### Targeting viperin to mitochondria leads to increased lipogenesis

**Figure 4:**
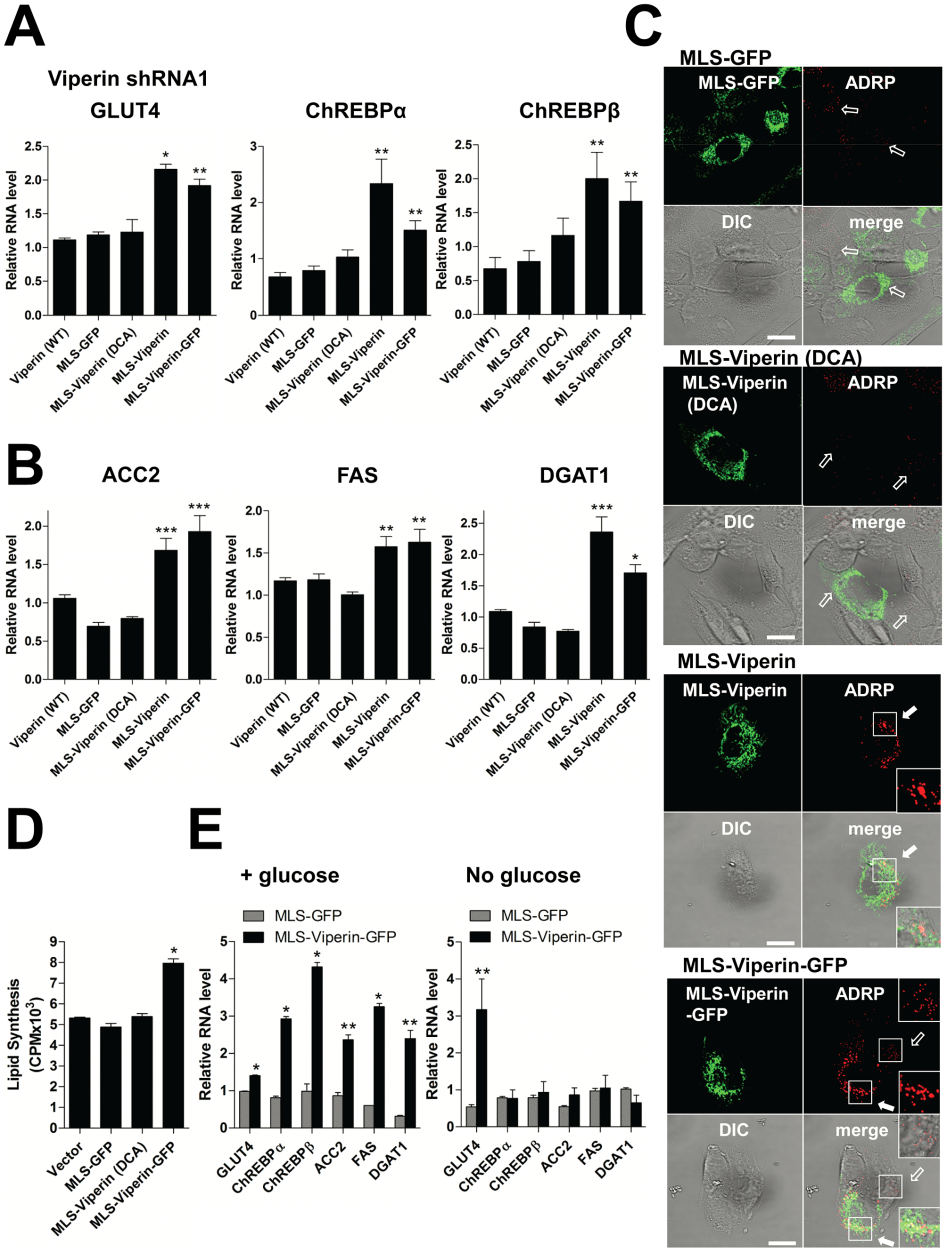
Targeting viperin to mitochondria leads to increased lipogenesis. The N-terminal 42 residue α-helical region of mouse viperin (WT), a mouse viperin-GFP chimera, or viperin (DCA) was replaced by the mitochondrial localization sequence (MLS) of the HCMV protein vMIA. The MLS was also directly fused to enhanced green fluorescent protein (EGFP) as a negative control (MLS-GFP). (**A** and **B**) mRNA levels of GLUT4 and ChREBPs (A) and lipogenic enzymes (**B**) in viperin knockdown HFtelo cells transiently expressing the indicated chimeric viperin proteins. The mRNA level in the cells expressing a control vector was set at 1. Data are represented as means ± SEM of triplicate samples and are representative of two individual experiments. *, *P*<0.01; **, *P*<0.05; ***, *P*<0.005. (**C**) LDs were monitored in viperin knockdown HFtelo cells transiently expressing the indicated chimeric viperin proteins. Cells were stained with ADRP and viperin antibodies. The filled arrows indicate the accumulated LDs in the transfected cells. The open arrows indicate the small basal LDs in the non-transfected or transfected cells. Scale bar, 20 µm. (**D**) Total lipid synthesis. Viperin knockdown HFtelo cells transiently expressing the indicated chimeric viperin proteins were labeled with [^14^C]-acetate for 3 hr. Total lipids from 5×10^4^ cells were extracted in chloroform-methanol and [^14^C] incorporation assessed. Data are presented as mean ± SEM of triplicate samples and are representative of two individual experiments. *, *P*<0.001. (**E**) The viperin knockdown HFtelo cells were transfected with the indicated constructs. At 1 day after transfection, the cells were transferred to serum-free medium in the presence and absence of glucose and incubated for 24 hr at 37°C. GLUT4, ChREBPs and lipogenic enzyme mRNA levels were measured. Data are represented as means ± SEM of triplicate samples and are representative of two individual experiments. *, *P*<0.005; **, *P*<0.05.

The reduction in cellular ATP levels and disruption of the actin cytoskeleton induced by vMIA-mediated transfer of viperin to mitochondria can be replicated in the absence of infection by directly targeting viperin using a mitochondrial localization sequence (MLS) [24]. The activity requires a functional Fe-S cluster binding site [24]. To determine if this is also true for the effects on lipogenesis, we used previously described chimeric mouse viperin constructs: MLS-viperin, in which the N-terminal amphipathic α-helix of viperin, responsible for its ER and LD association [39], [40], was replaced by the MLS of vMIA, and MLS-viperin (DCA) in which two cysteine residues (88 and 91) required for Fe-S cluster association were mutated to alanine [24]. We also used a fusion construct with the enhanced green fluorescent protein (EGFP) attached to MLS-viperin, and one with the MLS linked to EGFP directly. Transfected cells were purified by Streptavidin Microbeads using a biotinylated antibody to Thy1.1, also encoded in the construct and separated by an IRES. We examined the expression of GLUT4 and ChREBP (Figure 4A), the intracellular localization of ChREBP (Figure S3A), the expression of lipogenic enzymes (Figure 4B), the accumulation of LDs (Figure 4C), and total lipid synthesis using ^14^C-labeled acetate as a substrate (Figure 4D) in human viperin knockdown cells expressing the chimeric proteins. Expression of the mouse constructs is not affected by the human-specific shRNAs. In contrast to the cells expressing a control vector or MLS-GFP, the cells expressing MLS-viperin or MLS-viperin-GFP significantly increased GLUT4, ChREBP and lipogenic gene expression, showed nuclear localization of ChREBP, exhibited increased total lipid synthesis, and accumulated LDs (Figure 4A–4D and S3A), indicating that mitochondrial viperin directly induces GLUT4-mediated *de novo* lipogenesis. Cells expressing wild type viperin, which does not accumulate in mitochondria, or MLS-viperin (DCA) did not show the increase in mRNA levels, nor did they increase total lipid synthesis or accumulate LDs (Figure 4A–4D). This indicates that the viperin effects on lipid metabolism require mitochondrial localization and Fe-S cluster binding. Similar results were observed in viperin knockdown cells expressing a chimeric viperin protein with the N-terminal α-helix replaced by the MLS of Tom70, a host cellular mitochondrial protein (Figure S3B). This excludes the possibility that the MLS from vMIA was itself responsible for the effect. To determine whether the *de novo* lipogenesis observed was dependent on GLUT4-mediated glucose uptake we measured lipogenic enzyme transcripts in cells expressing the chimeric proteins in the presence or absence of glucose in the medium (Figure 4E). In the absence of glucose the cells expressing MLS-viperin-GFP had significantly increased GLUT4 mRNA content, but mRNA levels for ChREBP and the lipogenic enzymes were unchanged. We also examined lipogenesis in GLUT4 and ChREBP knockdown cells expressing the chimeric proteins (Figure S3C). The expression of ChREBP and lipogenic enzymes in GLUT4 knockdown cells expressing MLS-viperin-GFP was unaffected. However, GLUT4 expression was induced in ChREBP knockdown cells expressing MLS-viperin-GFP, while the expression of lipogenic enzymes was unaffected (Figure S3C). The data support a mechanism in which mitochondrial viperin enhances GLUT4 expression, increasing glucose uptake and thus ChREBP activation, which in turn activates *de novo* lipogenesis. Similar results were generated using viperin knockout murine embryonic fibroblasts (MEFs) expressing the chimeric proteins (Figure S4). In contrast to the cells expressing a control vector or MLS-GFP, cells expressing MLS-viperin-GFP exhibited significantly increased lipogenic gene expression (Figure S4A), accumulated LDs (Figure S4B), and increased total lipid synthesis (Figure S4C).

### Viperin interaction with TFP is responsible for the increased lipogenesis

**Figure 5:**
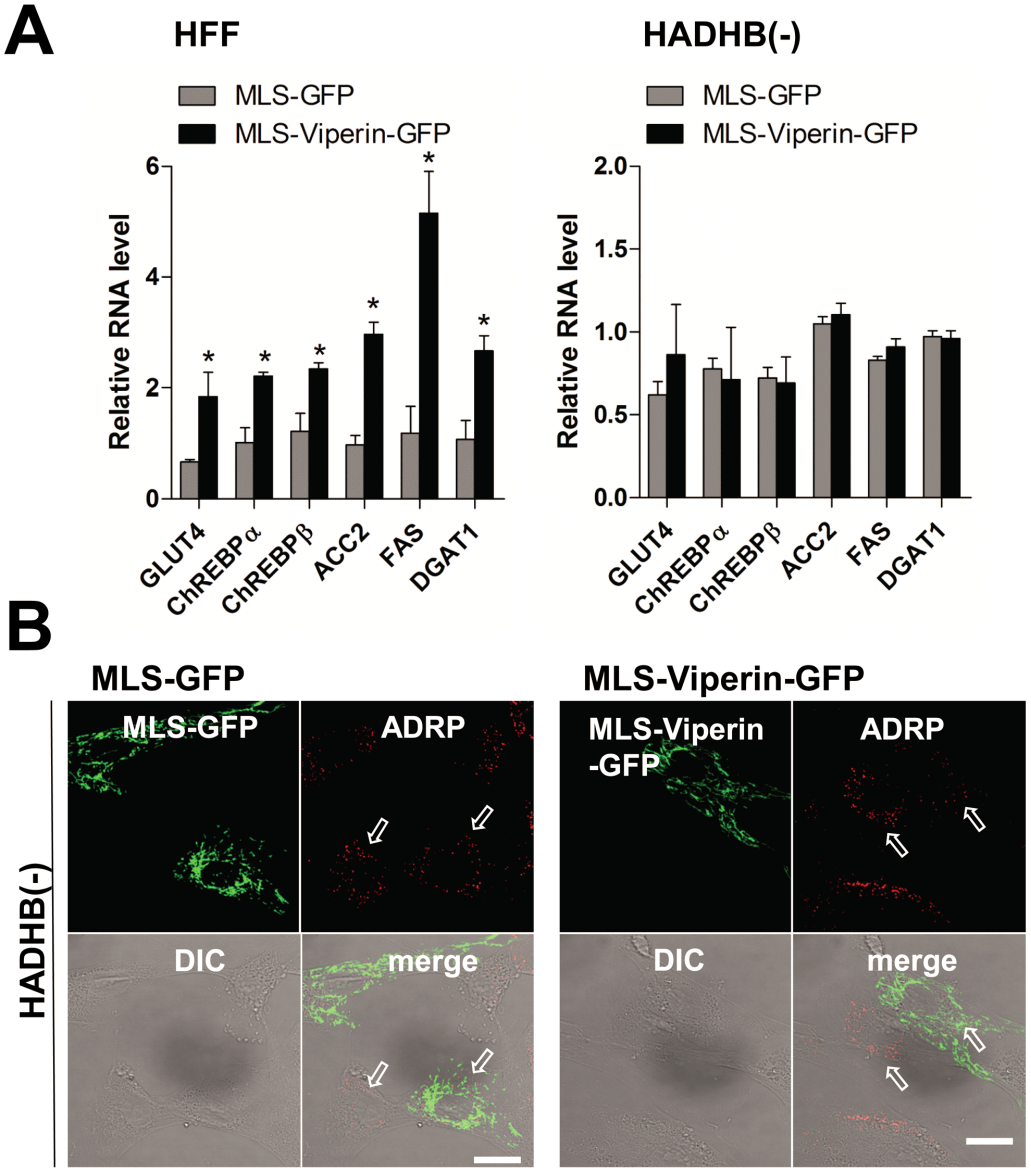
Viperin interaction with TFP is responsible for increased lipogenesis. (**A**) mRNA levels of GLUT4, ChREBPs and lipogenic enzymes in TFP β subunit deficient (HADHB (−)) cells transiently expressing the indicated chimeric viperin proteins were measured as described above. Data are represented as means ± SEM of triplicate samples and are representative of two individual experiments. *, *P*<0.05. (**B**) LDs were monitored in HADHB (−) cells transiently expressing the indicated chimeric viperin proteins as described above. The arrows indicate the small basal LDs in the non-transfected or transfected cells. Scale bar, 20 µm.

**Figure 6:**
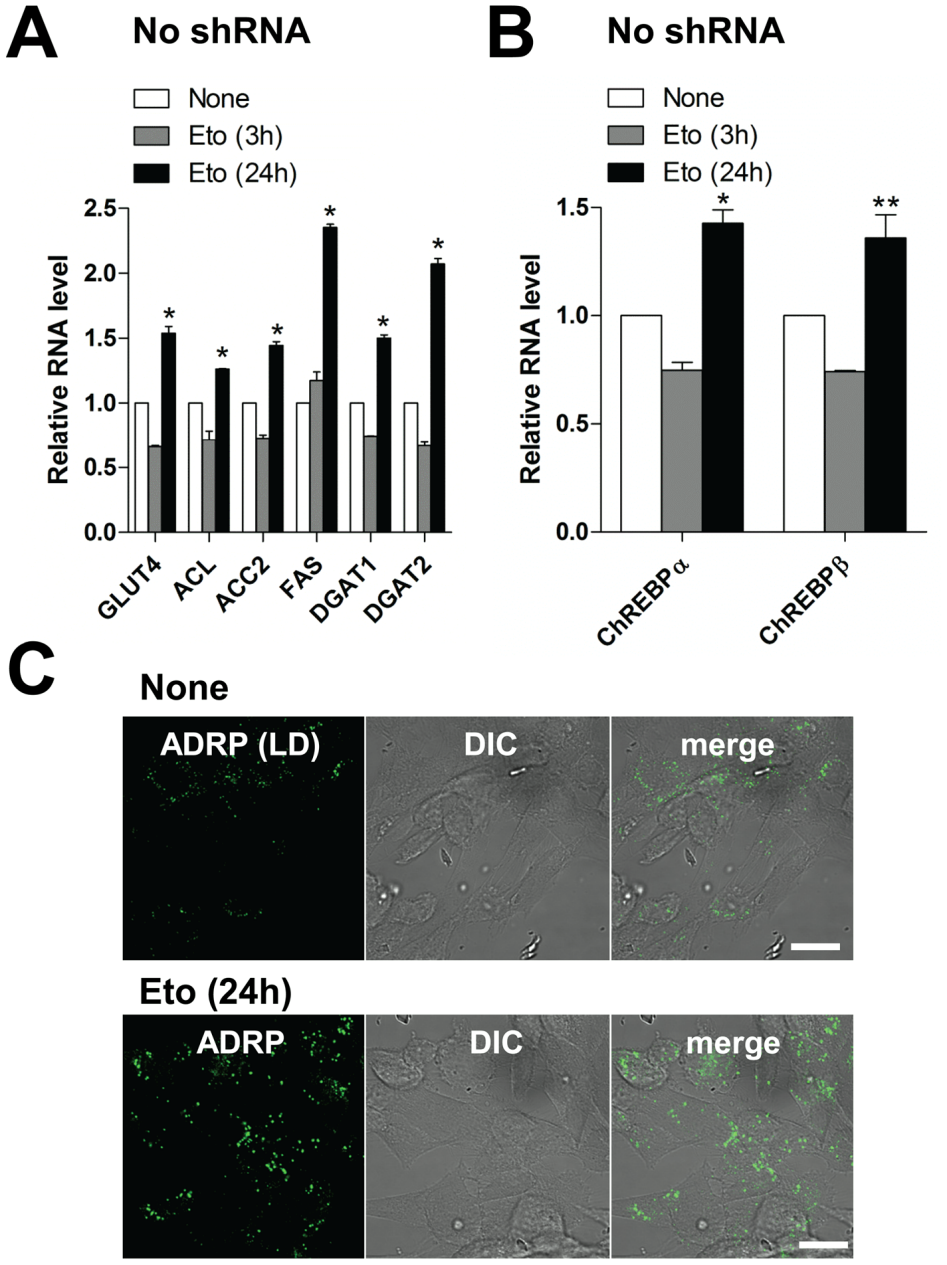
Inhibition of fatty acid β-oxidation induces lipogenesis. (**A** and **B**) mRNA levels of GLUT4 and lipogenic enzymes (**A**) and ChREBPs (**B**) in HFtelo cells treated with etomoxir (0.2 mM), an inhibitor of mitochondrial long chain fatty acid oxidation, for 3 or 24 hr. Data are represented as means ± SEM of triplicate samples and are representative of two individual experiments. *, *P*<0.005; **, *P*<0.05. (**C**) LDs were monitored in etomoxir-treated HFtelo cells. Cells were stained with ADRP and viperin antibodies. Scale bar, 20 µm.

Our previous work showed that viperin interaction with the mitochondrial enzyme TFP inhibits fatty acid β-oxidation [24]. To determine whether this interaction is responsible for the observed effects on lipid metabolism, we measured lipogenesis in human fibroblasts genetically deficient in the TFP β subunit (HADHB) transiently expressing each chimeric protein. In contrast to the results with wild type fibroblasts, lipogenesis in TFP-deficient fibroblasts expressing either MLS-viperin-GFP or Tom70MLS-viperin was unaffected (Figure 5 and S5). These results indicate that the viperin interaction with TFP is required for the enhanced lipid synthesis induced by HCMV infection, suggesting that GLUT4 activation and lipogenic enzyme stimulation are likely downstream of the inhibition of fatty acid β-oxidation. To address this we examined lipogenesis in cells treated with etomoxir, an inhibitor of mitochondrial long chain fatty acid oxidation (Figure 6). As anticipated, increased expression of GLUT4, ChREBP and lipogenic enzymes, as well as accumulation of LDs, were all observed in the etomoxir-treated cells. Taken together, the data indicate that the interaction of viperin with TFP and consequent inhibition of fatty acid β-oxidation is required for the induction of lipogenesis.

### The effects of HCMV infection on lipogenesis depend on the viperin-TFP interaction

**Figure 7:**
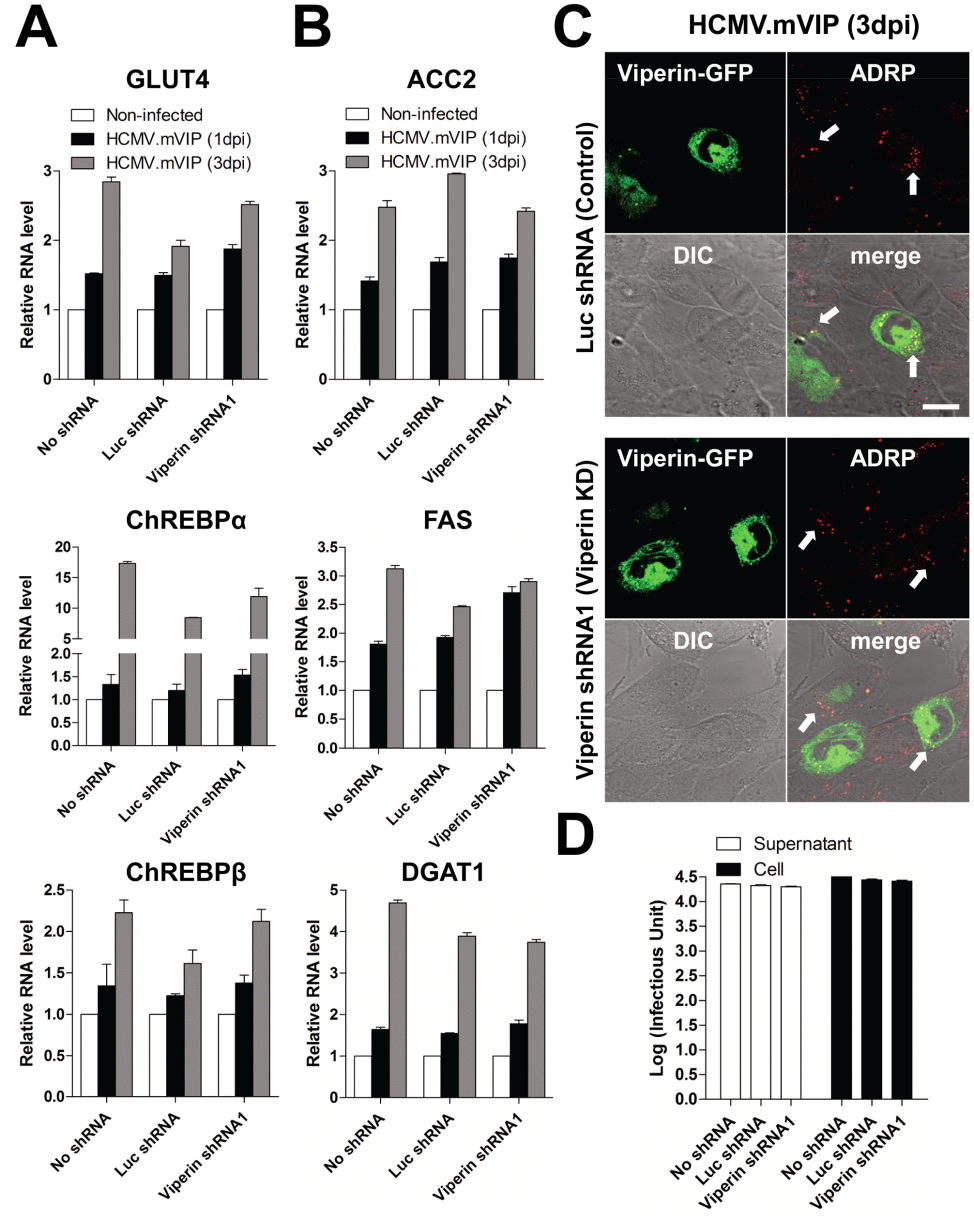
Specificity of the viperin effects on modulation of lipid metabolism in HCMV infection. (**A**–**C**) HFtelo cells stably expressing the indicated shRNAs were infected with recombinant HCMV.mVIP, in which the loci US7–US16 were replaced by doxycycline (Dox)-inducible mouse viperin-GFP, at an moi of 2 for 1 or 3 days. Dox (2 µg/ml) was added before infection. mRNA levels of GLUT4 and ChREBPs (**A**) and lipogenic enzymes (**B**) were measured. Data are presented as means ± SEM of triplicate samples and are representative of two individual experiments. Cells were stained with antibody specific to ADRP (**C**). The HCMV.mVIP-infected cells were detected by expression of viperin-GFP. The arrows indicate the accumulated LDs. Scale bar, 20 µm. (**D**) HFtelo cells expressing the indicated shRNAs were infected with HCMV.mVIP at an moi of 0.2, and supernatants and cells were harvested at 6 dpi. Dox (2 µg/ml) was added on day 0 and 3. Virus yield was quantified by a fluorescence-based virus infectivity assay. Data are presented as means ± SEM of duplicate samples and are representative of two individual experiments.

Finally, to control for the specificity of the viperin shRNA effects and confirm that viperin is involved in lipogenesis in the context of viral infection, we infected cells with a recombinant HCMV (HCMV.mVIP) in which the loci US7–US16, nonessential for in vitro replication, were replaced by mouse viperin-GFP under an inducible promoter such that expression could be enhanced by the addition of doxycycline, although some breakthrough transcription occurred in the absence of doxycycline [24]. Expression of mouse viperin-GFP upon HCMV.mVIP infection restored the wild type phenotype in human viperin knockdown cells in terms of expression of GLUT4, ChREBP and lipogenic enzymes (Figure 7A and B), accumulation of LDs (Figure 7C), formation of viral envelope (Figure S6), and production of infectious extracellular and intracellular virus (Figure 7D).

## Discussion

The effects of HCMV upon the metabolic status of infected cells reflect a commitment to macromolecular synthesis, including lipid biosynthesis, rather than generation of energy [4], [5], [6], [7]. Intracellular ATP levels actually decrease, and this has been postulated to induce disruption of the actin cytoskeleton that enhances viral replication [25], [26], [27], [28]. Lipogenesis is increased and LDs accumulate, interpreted to reflect the requirement for viral envelope [4], [5], [6], [7], [10], [11]. We previously showed that the decrease in ATP and cytoskeletal actin modulation is because viperin, induced by HCMV and translocated to mitochondria by vMIA, interacts with TFP and inhibits fatty acid β-oxidation [24]. We now show that these events are also responsible for increased lipogenesis. When viperin expression is prevented in HCMV-infected cells they do not exhibit the alterations of lipid metabolism normally observed and, although viral protein synthesis is unaffected and capsids are generated, envelope formation is impaired and the production of infectious virus is reduced. Furthermore, all of these features are restored when mouse viperin is expressed from an HCMV recombinant. In addition, infection by an HCMV mutant lacking vMIA, and therefore unable to transfer viperin to mitochondria, fails to induce lipogenesis. Targeting viperin to mitochondria induces these metabolic changes directly, but not when the cells lack functional TFP, and an activity of viperin mediated by Fe-S cluster binding is essential.

The cumulative data suggests that viperin-mediated inhibition of fatty acid β-oxidation reduces the generation of ATP and activates AMPK, which results in the induction of GLUT4 to increase glucose uptake. Activation of AMPK has been shown to be important for HCMV-mediated alterations in metabolism [9]. The increase in cytosolic glucose, in addition to inducing glycolysis, induces ChREBP, which, upon binding glucose, accumulates in the nucleus and activates the transcription of genes encoding lipogenic enzymes, enhancing lipid synthesis during HCMV infection. Interestingly, in HCMV-infected cells expressing viperin-specific shRNAs ChREBP translocation to the nucleus was indeed inhibited, but it was polarized rather than remaining diffuse and cytosolic (Fig. 1D). The localization is reminiscent of the localization of the complex virus assembly compartment that develops in HCMV-infected cells, but as HCMV infection causes multiple effects on cellular morphology the meaning of this observation is unclear. It is also conceivable that the apparent redistribution of ChREBP may an artifact since HCMV expresses an Fc receptor that localizes to the AC late in infection and binds non-specifically to polyclonal rabbit antibodies. This requires further analysis. Consistent with the role of GLUT4-mediated glucose import in inducing ChREBP activity, when viperin is targeted to mitochondria directly in cells cultured without glucose, GLUT4 transcription is still induced but ChREBP transcription is not; nor is the transcription of the lipogenic enzymes that depend on ChREBP expression. We do not know how rapidly these effects on metabolism begin after HCMV infection. Most measurements have been performed at day 1 post-infection or later. However, the kinetics of viperin and vMIA expression are very similar, and viperin substantially co-localizes with mitochondria after 24 hrs, so we might expect that these effects are initiated quite rapidly during the infection.

Lipid synthesis is induced by HCMV infection, and in cells lacking viperin expression this increased synthesis is not observed. Presumably because of this the increase in size and number of LDs induced by infection is also not seen in the viperin shRNA-expressing cells. These findings are precisely replicated in the same cells uninfected but expressing mitochondrially-targeted viperin. HCMV infection in the absence of viperin induction both eliminates the size increase and reduces the number of LDs, suggesting that initiating viral envelope formation consumes pre-existing lipids but without the viperin-mediated induction of lipogenesis they are not replaced. This results in a significant decrease in the production of infectious virus. Envelopment of HCMV proceeds by a complex process, involving initial envelopment in nuclear membrane that is shed in the cytoplasm. Secondary envelopment occurs by budding into the secretory pathway. Electron microscopy clearly showed that the number of enveloped particles formed in the secretory pathway was substantially reduced in the viperin knock down cells. However, the synthesis of viral proteins and the total number of viral particles, assessed by genome copy number was unaffected.

Although HCMV-induced cleavage of SREBP1 is independent of viperin expression, HCMV infection does not induce expression of lipogenic enzymes and lipid synthesis in the absence of viperin. There are two genes encoding SREBPs, SREBP1 and SREBP2, and SREBP1 exists in two forms, a and c, depending on the use of alternate promoters. This renders a simple interpretation difficult, but mice lacking SREBP1c do exhibit a reduction of some, but not all lipogenic enzyme transcripts in the liver [41]. Hepatic mRNA expression levels of lipogenic genes were also decreased in ChREBP knockout mice, resulting in a 65% reduction of fatty acid synthesis rates, despite normal mRNA levels of SREBP1c and cleavage of SREBP1 protein [12]. In the case of HCMV infection SREBP1 cleavage may be insufficient to substantially activate lipogenesis in the absence of ChREBP induction, even though preventing SREBP1 cleavage using an shRNA specific for the enzyme SCAP (SREBP-cleavage activating protein) inhibited lipid synthesis and HCMV growth [10], [11]. SREB1a also activates genes controlling cholesterol biosynthesis, which may be why inhibiting SREBP cleavage also affects HCMV replication. Thus there may be synergy between SREBP1 and ChREBP in ensuring efficient membrane generation. Nevertheless, the viperin-dependent induction of lipogenesis by ChREBP is clearly essential for a successful HCMV infection.

Overall, the data indicate that viperin is the key regulator of HCMV-induced modulation of lipid metabolism, and it may present a novel target for HCMV therapy. We are still left, however, with the vexed question of the normal function of viperin. Are there physiological circumstances in which a modification of lipogenesis mediated by viperin would be important, and if that is so, how does the molecule enter mitochondria to mediate this effect? Does this play a role in any of the inhibitory effects on other viruses that have been attributed to viperin? The answers to these questions await further experiments.

## Materials and Methods

### Cells, viruses, antibodies, and reagents

Human foreskin fibroblast (HFF) cells were purchased from Yale Skin Diseases Research Center Core. Telomerase-immortalized human fibroflast (HFtelo) cells were kindly provided by Dr. T. Shenk (Princeton University). Trifunctional protein (TFP) β subunit (HADHB) deficient fibroblasts (GM20265 and GM20266) were obtained from the Coriell Institute. MRC5 fibroblasts were kindly provided by Dr. E.S. Mocarski (Emory University). Murine embryonic fibroblasts (MEFs) used in the study were isolated from viperin (*Rsad2*) knockout C57BL/6 mice [24], [42] and immortalized by serial passages as described previously [43].

HCMV strain AD169 was kindly provided by Dr. W.J. Britt (University of Alabama at Birmingham). HCMV strain RVHB5 and HCMV mutant RVHB5ΔvMIA were provided by Dr. E.S. Mocarski (Emory University) [38]. Recombinant HCMV.mVIP used in the study was previously constructed utilizing a recombination strategy to insert Tet-on inducible mouse viperin-GFP fusion plasmid into the HCMV genome maintained in a bacterial artificial chromosome (BAC) [24].

HCMV-encoded proteins were detected with monoclonal antibodies (mAb). mAbs to MCP (*UL86*) (28-4), pp65 (*UL83*) (28-19), pp28 (*UL99*) (41-18), gB (*UL55*) (27-78) and IE-1 (*UL123*) (P63-27) were gifts from Dr. W.J. Britt (University of Alabama at Birmingham). mAb to viperin (aa 263-277) (MaP.VIP) was described previously [44], [45]. mAbs to adipose differentiation-related protein (ADRP) (Progen), SREBP1 (IgG-2A4, BD Pharmingen) and GRP 94 (Research Diagnostics) were used. Polyclonal rabbit Abs to ChREBP (Abcam) and actin (Santa Cruz Biotech) were also used. Goat anti-rabbit and anti-mouse Ig secondary Abs were purchased from Molecular Probes.

Caspase inhibitor I, ZVAD (OMe)-FMK (Calbiochem) and AMPK inhibitor, Compound C (Sigma) were used.

### AMPK activity assay

HFtelo cells stably expressing the indicated shRNAs were plated in duplicate and serum starved for 24 hr. Cells were infected with HCMV at a multiplicity of infection (moi) of 2. The cells were treated with DMSO or the AMPK inhibitor, Compound C (5 µM). At 2 dpi, the cells were harvested in lysis buffer (50 mM Tris-HCl, pH 7.4, 1% Triton X-100, 1 mM EGTA, protease inhibitor cocktail (+EDTA) (Roche), Phosphatase inhibitor cocktail (Roche) and 1 mM DTT). The cell lysates were assayed for AMPK activity using CycLex AMPK Kinase Assay Kit (MBL International Corporation). Absorbance of each sample was measured at wavelength of 450 nm using a Victor2 fluorometer (PerkinElmer).

### shRNA knockdown of viperin, GLUT4 and ChREBP

Previously generated HFtelo cells stably expressing two distinct short hairpin target sequences to viperin (Viperin shRNA1 and 2) and a negative control, a target sequence to luciferase (Luc shRNA) were used [24].

pGIPZ lentiviral plasmids with the short hairpin target sequences to GLUT4 and ChREBP were used (Open Biosystems). To increase knockdown efficiency, two lentiviral plasmids including different target sequences were combined: GLUT4 shRNA1 (clone ID: V3LHS_376729 and V3LHS_376733), GLUT4 shRNA2 (clone ID: V3LHS_376728 and V3LHS_376730), ChREBP shRNA1 (clone ID: V3LHS_334248 and V3LHS_334249) and ChREBP shRNA2 (clone ID: V3LHS_334249 and V3LHS_313472). HFtelo cells stably expressing the indicated shRNAs were generated as described previously [24]. Knockdown efficiency was assessed by western blot or RT-PCR analysis.

### Measurement of lipid synthesis

Lipid synthesis was measured as described [11], [46]. Briefly, HFtelo cells stably expressing the indicated shRNAs were plated in triplicate and serum starved for 24 hr. The cells were infected with HCMV at a multiplicity of infection (moi) of 2. At 2 hpi, cells were washed, re-fed with fresh serum-free DMEM, and incubated at 37°C for 2 days. For transient expression assays, viperin knockout MEFs were transfected with plasmids encoding chimeric proteins. At 1 day after transfection, the cells were washed, re-fed with fresh serum-free DMEM, and incubated at 37°C for 24 hr. The cells were then counted and incubated in fresh serum-free DMEM containing [1,2-^14^C] acetate (2 µCi/ml) and incubated at 37°C for 3 hr. Labeled cells were washed three times with ice-cold PBS and lysed in 0.3 ml of Triton X-100 (0.5% in H_2_O). Lipids were extracted by sequentially adding 0.75 ml of methanol, 0.75 ml of chloroform, 0.75 ml of chloroform, and 0.75 ml of H_2_O, with vortexing. Samples were centrifuged at 4,000 rpm for 15 min; 1.2 ml of organic phase (lower phase) was recovered and counted in a scintillation counter.

### Plasmids and transfections

The mouse wild type viperin cDNA construct of was generated by PCR amplification and then cloned into pMXs-IRES-Thy1.1. Chimeric viperin constructs were also generated in which residues 1–42 of WT viperin, WT viperin-GFP or mutant viperin (DCA, substitution of cysteine residues 88 and 91 to alanine residues) were replaced by the residues 1–34 (mitochondrial localization sequence, MLS) of vMIA and then cloned into pMXs-IRES-Thy1.1 to yield MLS-viperin, MLS-viperin-GFP or MLS-viperin (DCA), respectively. For the chimeric viperin construct Tom70-MLS-viperin, residues 1–42 of WT viperin were replaced by the MLS (residues 35–68) of human Tom70 and cloned into pMXs-IRES-Thy1.1. EGFP was also fused to residues 1–34 of vMIA (MLS-GFP) or residues 35–68 of Tom70 (Tom70-MLS-GFP). Plasmids were electroporated into viperin, GLUT4 and ChREBP knockdown HFtelo cells and TFP β subunit (HADHB) deficient HFF cells using a Nucleofector kit (Lonza). At 24 hr after transfection, the transfected cells were sorted by Streptavidin Microbeads (MACS Miltenyi Biotec) according to the manufacturer's instructions. The sorted cells were used for quantitative RT-PCR to measure the mRNA levels of lipogenic enzymes.

### Immunofluorescence

Lipid droplets (LDs) were monitored by immunofluorescence. The HCMV-infected or transfected fibroblasts were grown in 24-well tissue culture plates containing a 13-mm-diameter coverslip. The coverslips were harvested by first washing the cells with PBS and then fixing the cells for 45 min at room temperature in 3% paraformaldehyde freshly prepared in PBS. The coverslips were washed in PBS and permeabilized with 0.5% saponin in PBS for 10 min. The coverslips were then blocked with 0.2% Tween in PBS containing 10% normal goat serum for 20 min at room temperature, followed by the addition of the anti ADRP mAb, and incubated for 1 hr at room temperature. Following washing with 0.2% Tween in PBS, the coverslips were incubated with goat anti-mouse Ig secondary Ab conjugated to dye diluted in 0.2% Tween in PBS containing 2.5% normal goat serum for 1 hr at room temperature. The coverslips were washed three times, rinsed once in PBS and mounted with ProLong Gold Antifade reagent (Molecular Probes). The images were acquired with a Leica TCS SP2 confocal microscope.

### Electron microscopy

HFtelo cells stably expressing the indicated shRNAs were infected with HCMV or recombinant HCMV.mVIP at an moi of 2 and examined by electron microscopy at 7 dpi as described previously [45], [47]. Briefly, the cells were fixed with 2.5% glutaraldehyde and post-fixed with 1% osmium tetroxide. Cells were *en bloc* stained with 2% uranyl acetate, dehydrated, infiltrated, and embedded in Epon. Sixty nanometer sections were stained with lead citrate and examined using a Tecnai 12 Biotwin electron microscope.

### Immunoblot analysis

Cleavage of SREBP1 was assayed by immunoblot as described [10]. Cells were washed with PBS and solubilized in disruption buffer (50 mM Tris (pH 7.4), 2% SDS, 5% 2-mercaptoethanol, 2.75% sucrose) containing proteinase inhibitors. The extracts were boiled for 5 min, and centrifuged at 14,000 *g* for 3 min. Supernatants were collected and boiled in reducing sample buffer. The supernatants were separated on 8% SDS-PAGE gels and transferred to PVDF membranes (Millipore). The immunoblots were blocked in 5% skim milk, 0.05% Tween in PBS and incubated with mAb against SREBP1, probed with anti-mouse Ig horseradish-conjugated secondary antibody, followed by incubation with enhanced chemiluminescence (ECL) reagents (Pierce).

HCMV proteins were detected by immunoblotting. Cell pellets were lysed in 1% Triton X-100 in TBS containing proteinase inhibitors. Supernatants of lysates were collected and mixed with reducing sample buffer. The supernatants were separated on 8% or 10% SDS-PAGE gels. The immunoblots were probed with the indicated antibodies as described above.

### RNA extraction, cDNA preparation, and quantitative real-time PCR

Cells were collected and total RNA extracted using the RNeasy Mini kit (Qiagen). cDNA synthesis was performed with 1–2 µg RNA using AffinityScript Multi Temperature cDNA synthesis kit according to the manufacturer's instructions (Stratagen). The cDNA obtained from cells was quantified by real-time quantitative PCR (Q-PCR) using SYBR Green (Applied Biosystems) on Stratagene Mx3000P QPCR system. The following primers were used; β actin, GCTCCGGCATGTGCAA (Fwd) and AGGATCTTCATGAGGTAGT (Rev); GLUT4, GGAGCTGGTGTGGTCAACACA (Fwd) and GGAGCAGAGCCACAGTCATCA (Rev); ACL, TGTAACAGAGCCAGGAACCC (Fwd) and CTGTACCCCAGTGGCTGTTT (Rev); ACC2, GACCACAGGTGAAGCTGAGA (Fwd) and GTGTTCCCGTCCCCTCTTC (Rev); FAS, AGGCTGAGACGGAGGCCATA (Fwd) and AAAGCTCAGCTCCTGGCGGT (Rev); DGAT1, TCGCCTGCAGGATTCTTTAT (Fwd) and GCATCACCACACACCAGTTC (Rev); DGAT2, TCACCTGGCTCAATAGGTCCA (Fwd) and CCAGCAATCAGTGCAGAATATG (Rev); ChREBPα, AGTGCTTGAGCCTGGCCTAC (Fwd) and TTGTTCAGGCGGATCTTGTC (Rev); ChREBPβ, AGCGGATTCCAGGTGAGG (Fwd) and TTGTTCAGGCGGATCTTGTC (Rev). The Q-PCR was performed in triplicate for each sample. Quantitation was performed by the comparative Ct (2–[delta][delta]Ct) method. The Ct value for each sample was normalized by the value for β actin gene. Three independent experiments were analyzed statistically for differences in the mean values, and *P* values are indicated in the figures.

The viral genome copy number was also quantified by real-time PCR as described previously [47], [48]. Viral DNA were isolated by QIAamp DNA blood kit (Qiagen). The following primers, which amplify a segment of the HCMV *UL55* gene were used; ACGTGAAGGAATCGCCAGGA (Fwd) and AGTTCCAGTACCCTGAAGTC (Rev).

### Virus replication assay

HFFs or HFtelo cells stably expressing the indicated shRNA were infected with HCMV or recombinant HCMV (HCMV.mVIP) at an moi of 0.2 for 2 hr, washed, and cultured in normal medium or medium supplemented with doxycycline. Supernatants and cells were harvested at 6 dpi, and virus yield was measured by a fluorescence-based virus infectivity assay [49].

### Statistical analyses

Results from all studies were compared with unpaired two-tailed Student's *t* test using GraphPad Prism 4 software. *P* values less than 0.05 were considered significant.

## Supporting Information

Figure S1 Viperin expression is necessary for HCMV-induced modulation of metabolism. HFtelo cells stably expressing no shRNA, control luciferase (Luc) shRNA or viperin shRNAs were infected with HCMV at an moi of 2 for the indicated days. (**A**) Viperin knockdown efficiency was analyzed by immunoblot using anti-viperin antibody (MaP.VIP). Grp94 served as a protein loading control. (**B**) Glycolysis. At 1 dpi, the cells were transferred to glucose and glutamine-free medium, incubated at 37° for 1 hr and glycolysis assessed by measuring the extracellular acidification rate (ECAR), using an XF Extracellular Flux Analyzer (Seahorse Bioscience). Data are presented as mean ± SEM of duplicate samples and are representative of two individual experiments.
(TIF)

Figure S2 Mitochondrial viperin induces *de novo* GLUT4 and ChREBP-mediated lipogenesis during HCMV infection. (**A**) Whole cell extracts were prepared from the infected cells. SREBP1 was detected by immunoblot using a specific antibody (IgG-2A4). Actin served as a protein-loading control. SREBP1-P, SREBP1 precursor; SREBP1-M, cleaved mature form of SREBP1. (**B**) HFtelo cells stably expressing two distinct GLUT4 shRNAs or ChREBP shRNAs were infected with HCMV at m.o.i. of 2 for 3 day. The knockdown efficiency was analyzed by RT-PCR. Actin served as a loading control. (**C**) GLUT4, ChREBP and lipogenic enzyme mRNA levels in HFtelo cells expressing luciferase (Luc), GLUT4 or ChREBP shRNAs after HCMV infection at an moi of 2 for 1 or 3 days. Total RNA was isolated, and the mRNA levels were measured by quantitative RT-PCR and normalized to β actin mRNA. Data are presented as means ± SEM of triplicate samples and are representative of two individual experiments. GLUT4, glucose transporter 4; FAS, fatty acid synthase; DGAT1, diacylglycerol acyltransferases 1. (**D**) MRC5 fibroblasts were infected with wild type RVHB5 strain of HCMV (HCMV) or mutant RVHB5 lacking vMIA (ΔvMIA-HCMV) at m.o.i. of 2 for 1 day in the presence of the caspase inhibitor ZVAD-FMK (50 µM). Cells were stained with anti-viperin (MaP.VIP) and anti-calnexin (ER marker) antibodies, antibodies to the HCMV protein pp65 to identify infected cells, and Mitotracker Red to visualize mitochondria. Scale bar, 10 µm. (**E**) MRC5 fibroblasts were infected with HCMV or ΔvMIA-HCMV at m.o.i. of 2 for 1 or 3 days in the absence and presence of ZVAD-FMCK5 (50 µM). Total RNA was isolated, and the mRNA levels were measured by quantitative RT-PCR and normalized to β actin mRNA. Data are presented as means ± SEM of triplicate samples and are representative of two individual experiments.
(TIF)

Figure S3 Targeting viperin to mitochondria induces *de novo* lipogenesis. (**A**) The N-terminal 42 residue α-helical region of the mouse viperin-GFP chimera was replaced by the MLS of the HCMV protein vMIA. The MLS was also directly fused to enhanced green fluorescent protein (EGFP) as a negative control (MLS-GFP). ChREBP localization in viperin knockdown HFtelo cells transiently expressing the indicated chimeric viperin proteins. Cells were stained with antibody specific to ChREBP. The filled arrows indicate ChREBP localized in the nucleus, and the open arrows indicate ChREBP localized in the cytoplasm. Scale bar, 20 µm. (**B**) The N-terminal 42 residue α-helical region of mouse viperin was replaced by the mitochondrial localization sequence (MLS) of Tom70, a host cellular mitochondrial protein. mRNA levels of lipogenic enzymes in viperin knockdown HFtelo cells transiently expressing the indicated chimeric viperin proteins. Data are represented as means ± SEM of triplicate samples and are representative of two individual experiments. *, *P*<0.05. (**C**) mRNA levels of GLUT4, ChREBP and lipogenic enzymes in GLUT4 or ChREBP knockdown HFtelo cells transiently expressing the indicated chimeric viperin proteins. The mRNA level in the cells expressing a control vector was set at 1. Data are represented as means ± SEM of triplicate samples and are representative of two individual experiments. *, *P*<0.05.
(TIF)

Figure S4 Targeting viperin to mitochondria leads to increased lipogenesis in viperin knockout MEFs. The N-terminal 42 residue α-helical region of mouse viperin (WT), a mouse viperin-GFP chimera, or viperin (DCA) was replaced by the mitochondrial localization sequence (MLS) of the HCMV protein vMIA. The MLS was also directly fused to enhanced green fluorescent protein (EGFP) as a negative control (MLS-GFP). (**A**) mRNA levels of lipogenic enzymes in viperin knockout MEFs (MEF-Viperin KO) transiently expressing the indicated chimeric viperin proteins. The mRNA level in the cells expressing a control vector was set at 1. Data are represented as means ± SEM of triplicate samples and are representative of two individual experiments. *, *P*<0.005. (**B**) LDs were monitored in viperin knockout MEFs transiently expressing the indicated chimeric viperin proteins. Cells were stained co-stained with ADRP and viperin antibodies. The filled arrows indicate the accumulated LDs in the transfected cells. The open arrows indicate the small basal LDs in the non-transfected or transfected cells. Scale bar, 20 µm. (**C**) Total lipid synthesis. Viperin knockout MEFs transiently expressing the indicated chimeric viperin proteins were labeled with [^14^C]-acetate for 3 hr. Total lipids from 5×10^4^ cells were extracted and [^14^C] incorporation assessed. Data are presented as mean ± SEM of triplicate samples and are representative of two individual experiments. *, *P*<0.001.
(TIF)

Figure S5 Viperin interaction with TFP is responsible for increased lipogenic enzyme gene expression. mRNA levels of lipogenic enzymes in TFP β subunit deficient (HADHB (−)) cells transiently expressing the indicated chimeric viperin proteins were measured as described above. Data are represented as means ± SEM of triplicate samples and are representative of two individual experiments. *, *P*<0.05.
(TIF)

Figure S6 Specificity of the viperin effects on formation of HCMV viral envelope. Transmission electron micrographs of recombinant HCMV.mVIP-infected HFtelo cells stably expressing the indicated shRNAs. Monolayers were infected with recombinant HCMV.mVIP at an moi of 2 and processed for EM at 7 dpi. Dox (2 µg/ml) was added on day 0 and 3. Multiple frames from each sample were imaged and photographed. Particles from a representative cell are shown. White and black arrows indicate non-enveloped particles and enveloped particles, respectively. Cyto, cytoplasm. Scale bar, 1 µm. The numbers of enveloped and non-enveloped particles were counted in each frame and calculated as a ratio of enveloped particles to total particles in the cytoplasm of infected cells. The graphs indicate the mean percent of the enveloped particles per total 10–30 particles in each frame (from >10 cells of each sample) ± SEM.
(TIF)
