# Supplementary figures and images for: Viperin Regulates Cellular Lipid Metabolism during Human Cytomegalovirus Infection
Source: PLoS Pathog. 2013 Aug 1;9(8):e1003497. doi: 10.1371/journal.ppat.1003497 (PMC3731232; doi:10.1371/journal.ppat.1003497)

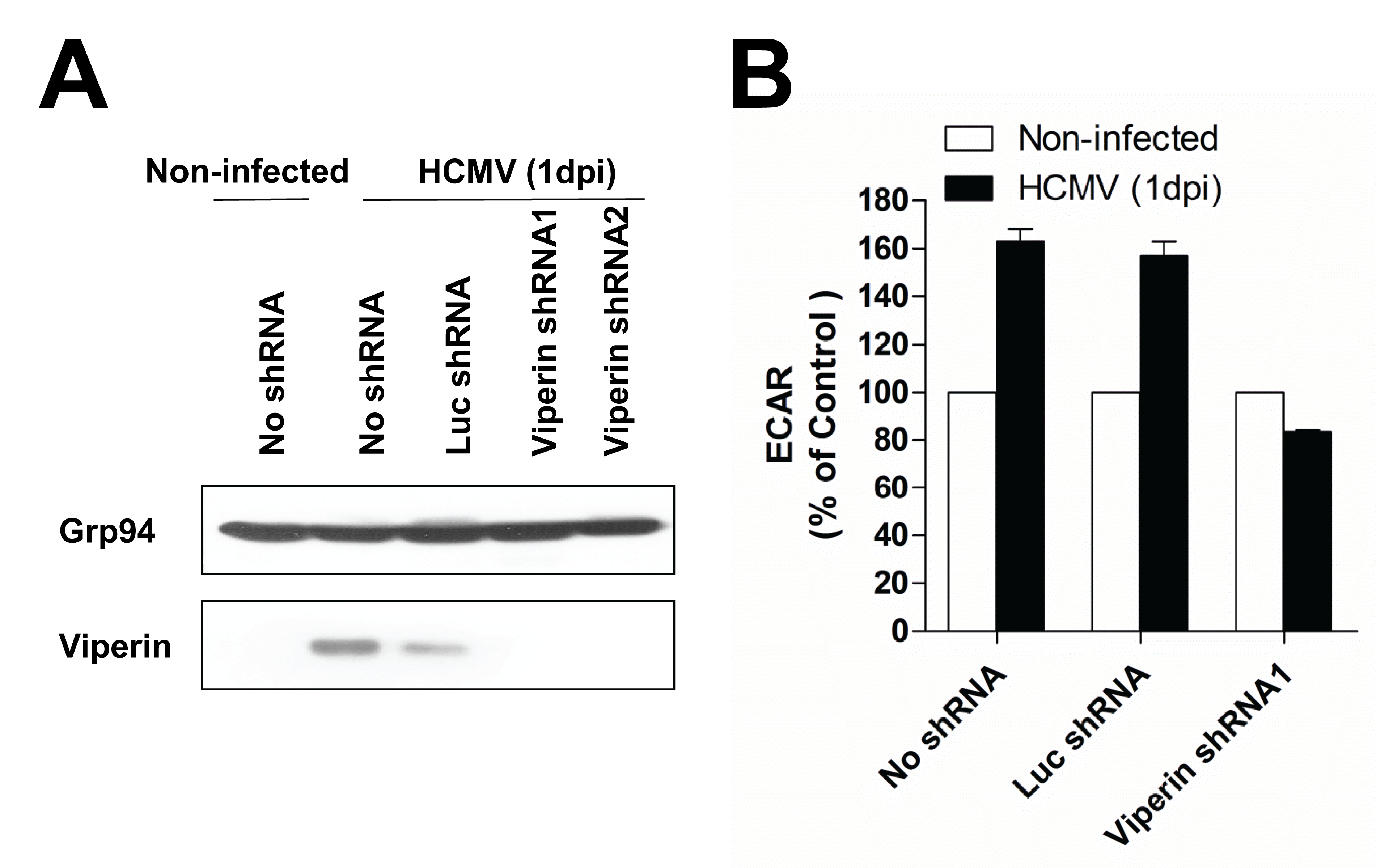

Supplement: Figure S1 — Viperin expression is necessary for HCMV-induced modulation of metabolism. HFtelo cells stably expressing no shRNA, control luciferase (Luc) shRNA or viperin shRNAs were infected with HCMV at an moi of 2 for the indicated days. (A) Viperin knockdown efficiency was analyzed by immunoblot using anti-viperin antibody (MaP.VIP). Grp94 served as a protein loading control. (B) Glycolysis. At 1 dpi, the cells were transferred to glucose and glutamine-free medium, incubated at 37° for 1 hr and glycolysis assessed by measuring the extracellular acidification rate (ECAR), using an XF Extracellular Flux Analyzer (Seahorse Bioscience). Data are presented as mean ± SEM of duplicate samples and are representative of two individual experiments. (TIF) [file ppat.1003497.s001.tif]

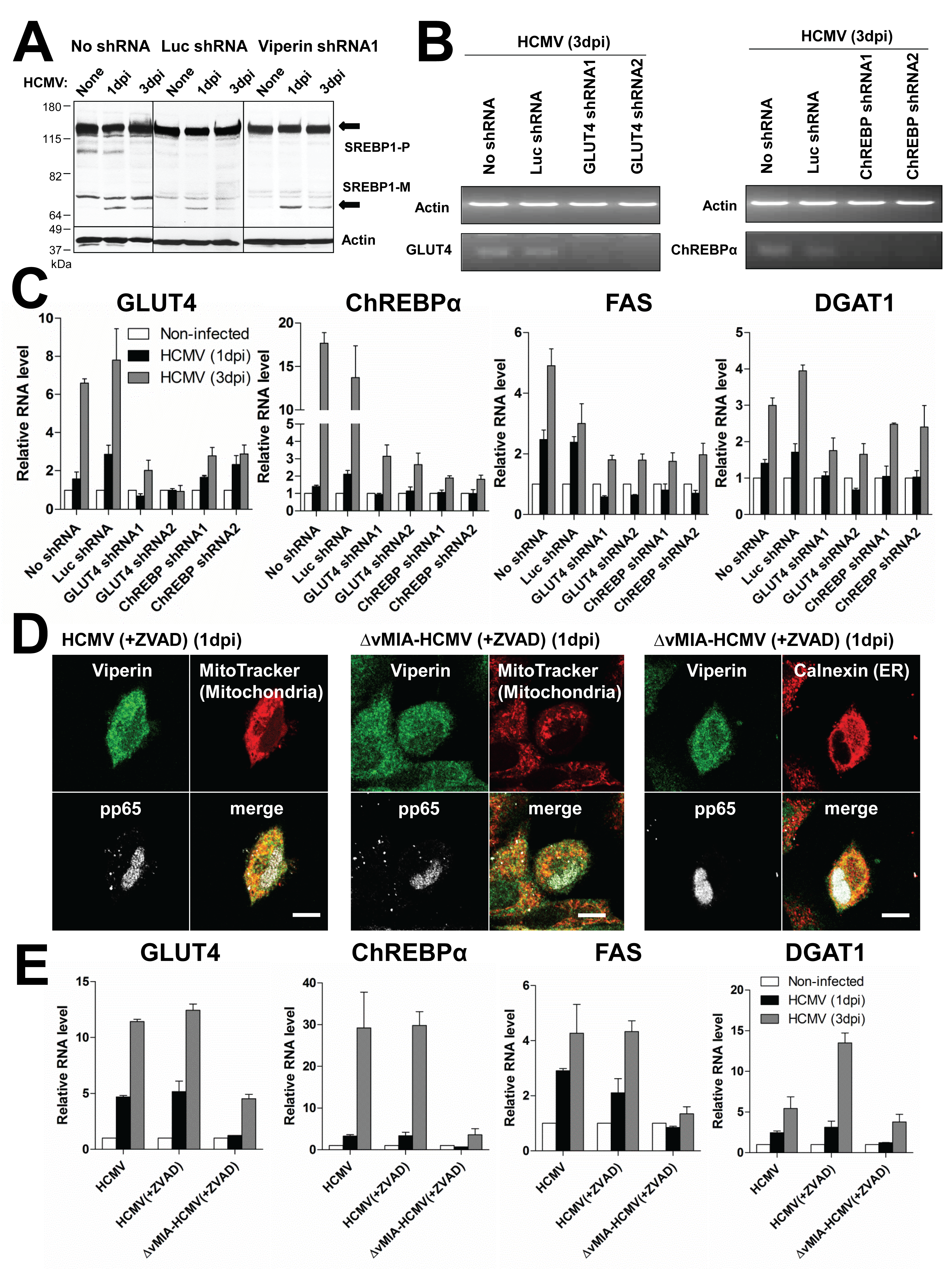

Supplement: Figure S2 — Mitochondrial viperin induces de novo GLUT4 and ChREBP-mediated lipogenesis during HCMV infection. (A) Whole cell extracts were prepared from the infected cells. SREBP1 was detected by immunoblot using a specific antibody (IgG-2A4). Actin served as a protein-loading control. SREBP1-P, SREBP1 precursor; SREBP1-M, cleaved mature form of SREBP1. (B) HFtelo cells stably expressing two distinct GLUT4 shRNAs or ChREBP shRNAs were infected with HCMV at m.o.i. of 2 for 3 day. The knockdown efficiency was analyzed by RT-PCR. Actin served as a loading control. (C) GLUT4, ChREBP and lipogenic enzyme mRNA levels in HFtelo cells expressing luciferase (Luc), GLUT4 or ChREBP shRNAs after HCMV infection at an moi of 2 for 1 or 3 days. Total RNA was isolated, and the mRNA levels were measured by quantitative RT-PCR and normalized to β actin mRNA. Data are presented as means ± SEM of triplicate samples and are representative of two individual experiments. GLUT4, glucose transporter 4; FAS, fatty acid synthase; DGAT1, diacylglycerol acyltransferases 1. (D) MRC5 fibroblasts were infected with wild type RVHB5 strain of HCMV (HCMV) or mutant RVHB5 lacking vMIA (ΔvMIA-HCMV) at m.o.i. of 2 for 1 day in the presence of the caspase inhibitor ZVAD-FMK (50 µM). Cells were stained with anti-viperin (MaP.VIP) and anti-calnexin (ER marker) antibodies, antibodies to the HCMV protein pp65 to identify infected cells, and Mitotracker Red to visualize mitochondria. Scale bar, 10 µm. (E) MRC5 fibroblasts were infected with HCMV or ΔvMIA-HCMV at m.o.i. of 2 for 1 or 3 days in the absence and presence of ZVAD-FMCK5 (50 µM). Total RNA was isolated, and the mRNA levels were measured by quantitative RT-PCR and normalized to β actin mRNA. Data are presented as means ± SEM of triplicate samples and are representative of two individual experiments. (TIF) [file ppat.1003497.s002.tif]

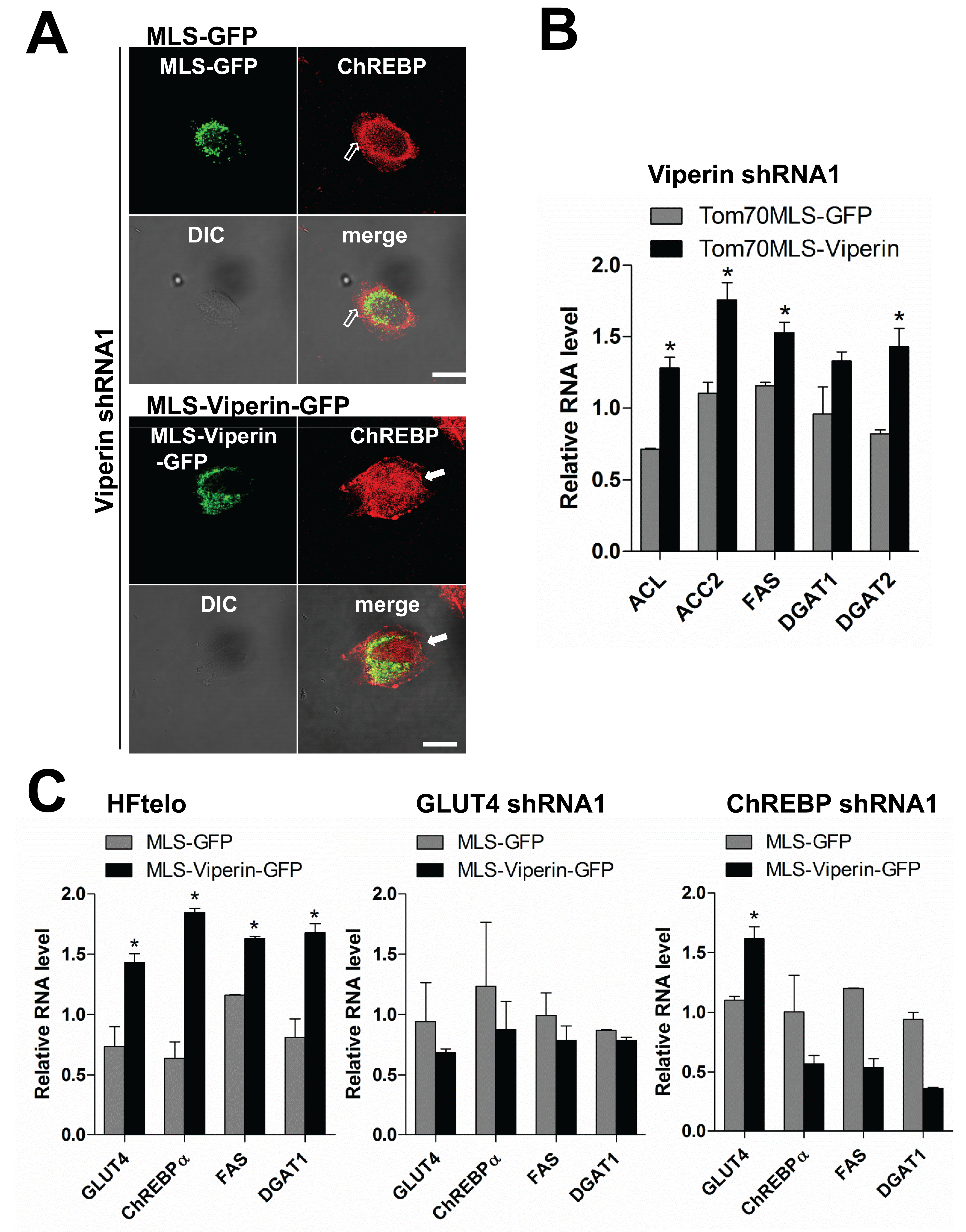

Supplement: Figure S3 — Targeting viperin to mitochondria induces de novo lipogenesis. (A) The N-terminal 42 residue α-helical region of the mouse viperin-GFP chimera was replaced by the MLS of the HCMV protein vMIA. The MLS was also directly fused to enhanced green fluorescent protein (EGFP) as a negative control (MLS-GFP). ChREBP localization in viperin knockdown HFtelo cells transiently expressing the indicated chimeric viperin proteins. Cells were stained with antibody specific to ChREBP. The filled arrows indicate ChREBP localized in the nucleus, and the open arrows indicate ChREBP localized in the cytoplasm. Scale bar, 20 µm. (B) The N-terminal 42 residue α-helical region of mouse viperin was replaced by the mitochondrial localization sequence (MLS) of Tom70, a host cellular mitochondrial protein. mRNA levels of lipogenic enzymes in viperin knockdown HFtelo cells transiently expressing the indicated chimeric viperin proteins. Data are represented as means ± SEM of triplicate samples and are representative of two individual experiments. *, P<0.05. (C) mRNA levels of GLUT4, ChREBP and lipogenic enzymes in GLUT4 or ChREBP knockdown HFtelo cells transiently expressing the indicated chimeric viperin proteins. The mRNA level in the cells expressing a control vector was set at 1. Data are represented as means ± SEM of triplicate samples and are representative of two individual experiments. *, P<0.05. (TIF) [file ppat.1003497.s003.tif]

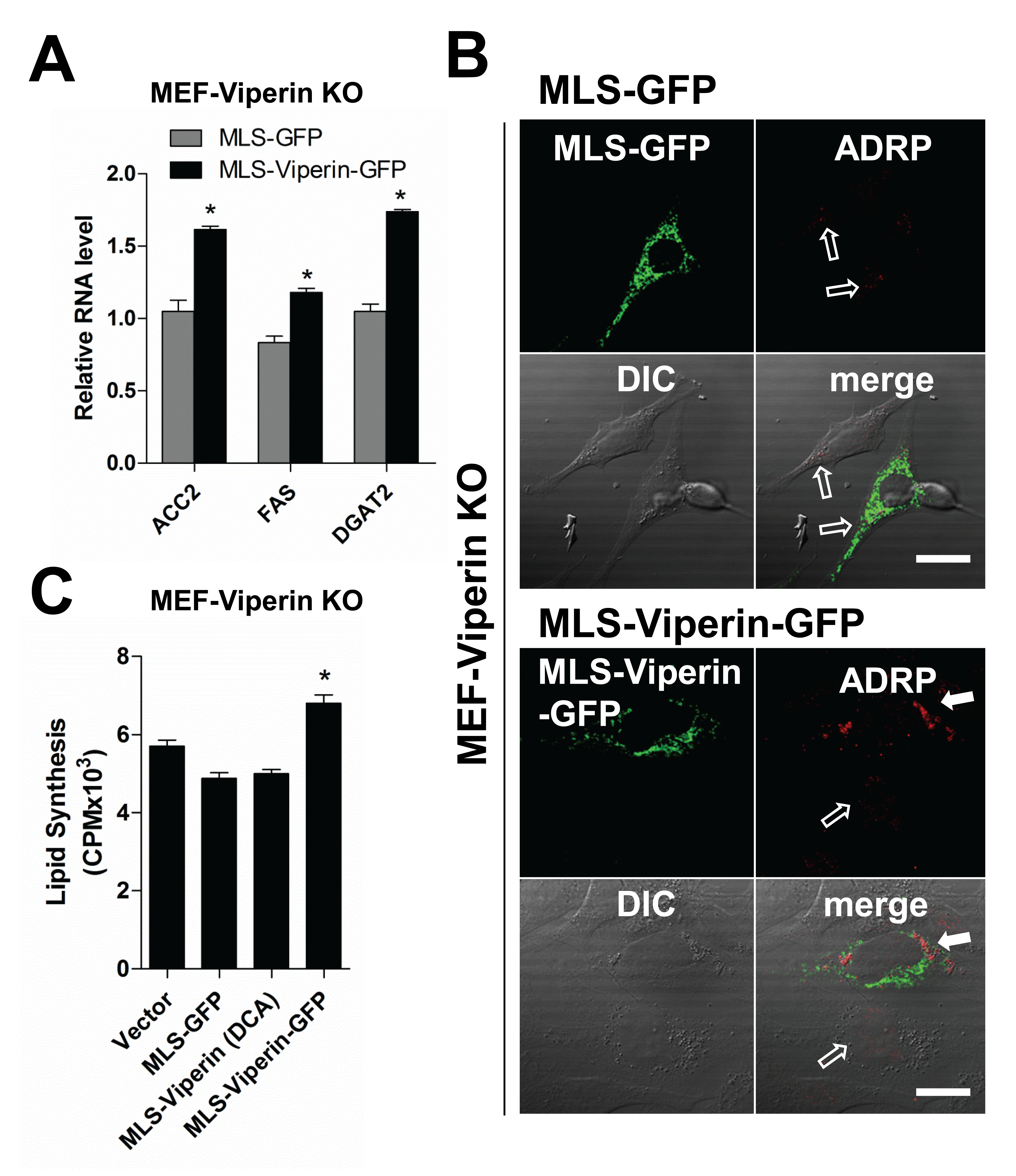

Supplement: Figure S4 — Targeting viperin to mitochondria leads to increased lipogenesis in viperin knockout MEFs. The N-terminal 42 residue α-helical region of mouse viperin (WT), a mouse viperin-GFP chimera, or viperin (DCA) was replaced by the mitochondrial localization sequence (MLS) of the HCMV protein vMIA. The MLS was also directly fused to enhanced green fluorescent protein (EGFP) as a negative control (MLS-GFP). (A) mRNA levels of lipogenic enzymes in viperin knockout MEFs (MEF-Viperin KO) transiently expressing the indicated chimeric viperin proteins. The mRNA level in the cells expressing a control vector was set at 1. Data are represented as means ± SEM of triplicate samples and are representative of two individual experiments. *, P<0.005. (B) LDs were monitored in viperin knockout MEFs transiently expressing the indicated chimeric viperin proteins. Cells were stained co-stained with ADRP and viperin antibodies. The filled arrows indicate the accumulated LDs in the transfected cells. The open arrows indicate the small basal LDs in the non-transfected or transfected cells. Scale bar, 20 µm. (C) Total lipid synthesis. Viperin knockout MEFs transiently expressing the indicated chimeric viperin proteins were labeled with [14C]-acetate for 3 hr. Total lipids from 5×10^4^ cells were extracted and [14C] incorporation assessed. Data are presented as mean ± SEM of triplicate samples and are representative of two individual experiments. *, P<0.001. (TIF) [file ppat.1003497.s004.tif]

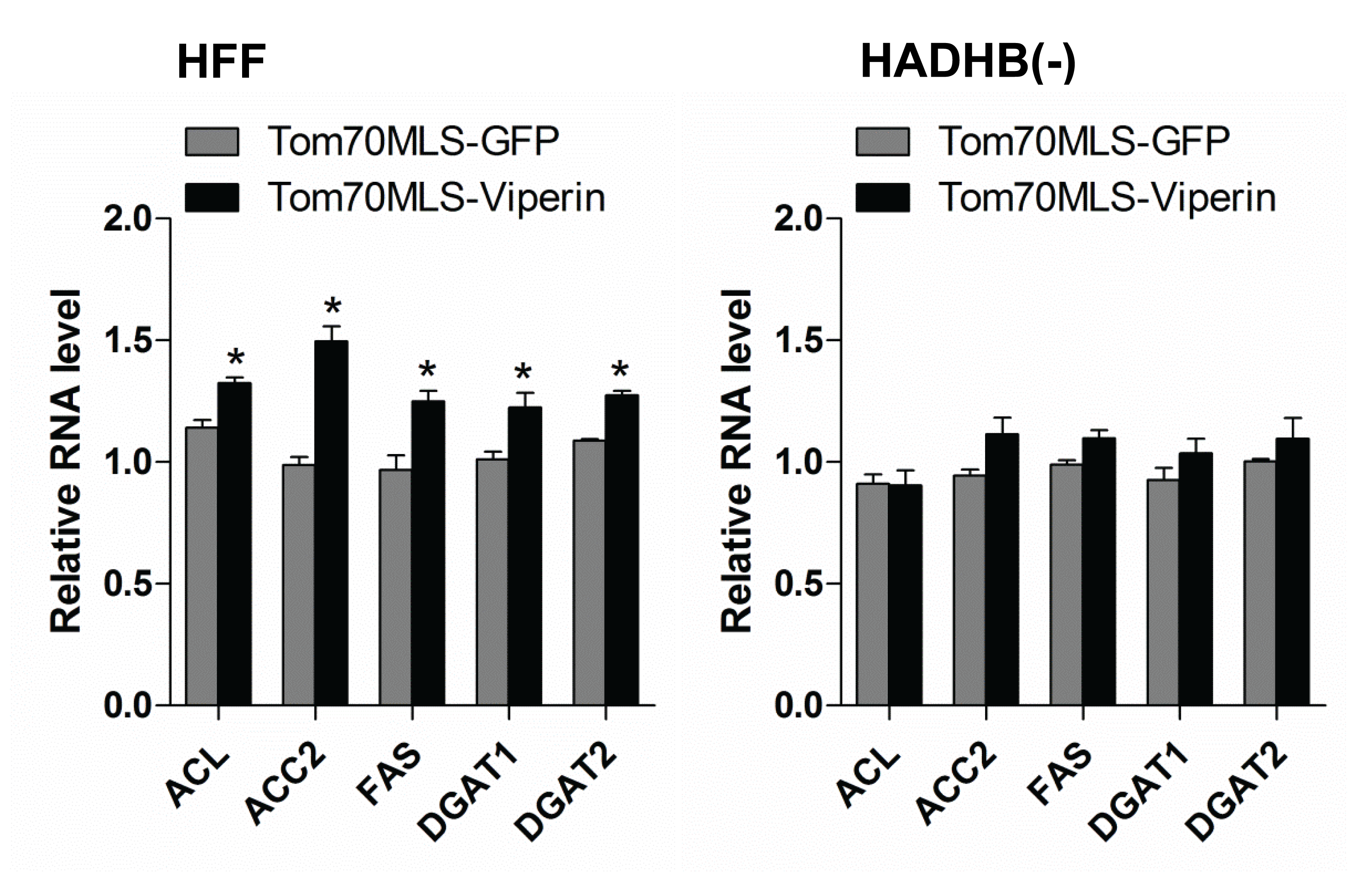

Supplement: Figure S5 — Viperin interaction with TFP is responsible for increased lipogenic enzyme gene expression. mRNA levels of lipogenic enzymes in TFP β subunit deficient (HADHB (−)) cells transiently expressing the indicated chimeric viperin proteins were measured as described above. Data are represented as means ± SEM of triplicate samples and are representative of two individual experiments. *, P<0.05. (TIF) [file ppat.1003497.s005.tif]

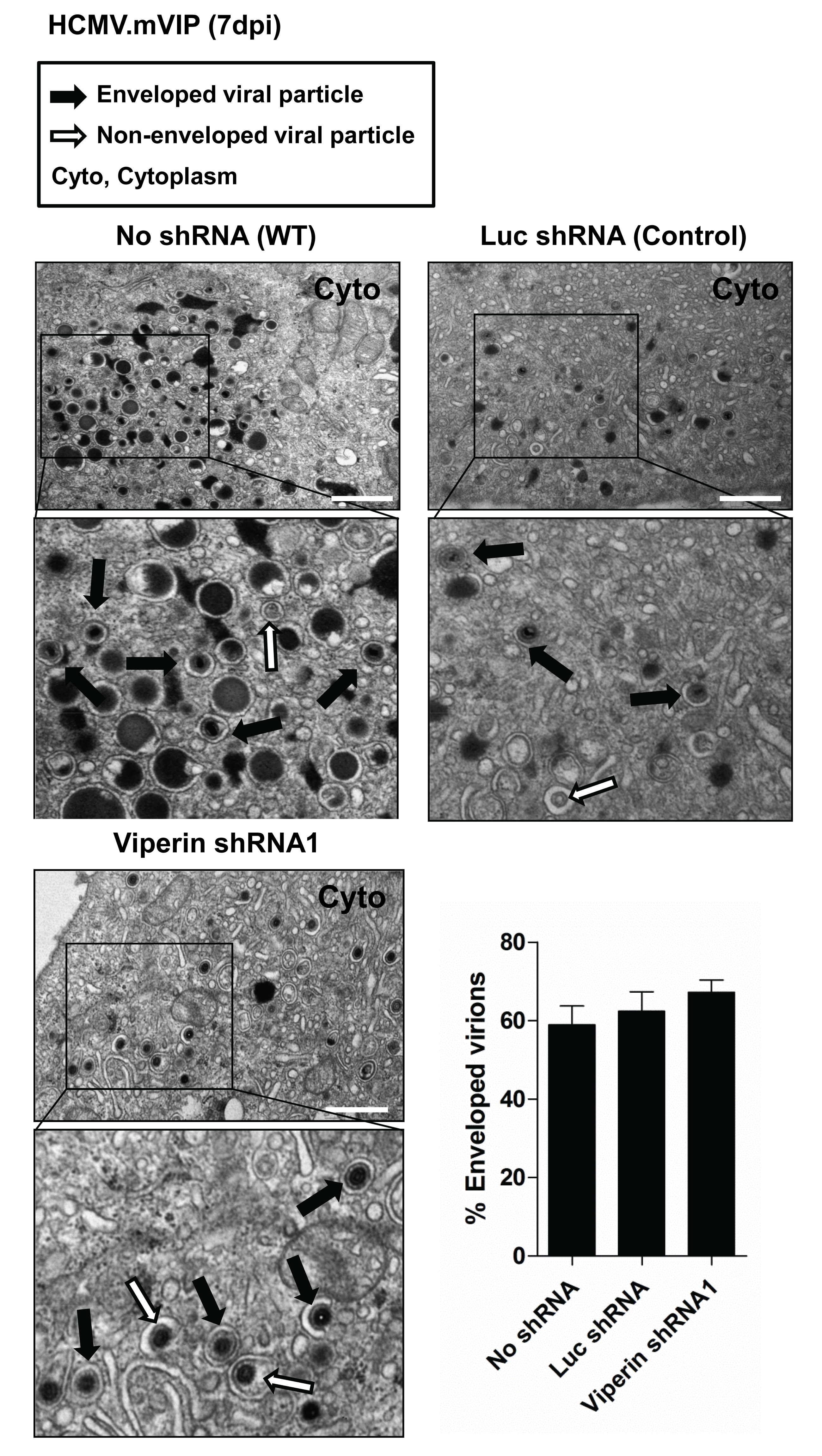

Supplement: Figure S6 — Specificity of the viperin effects on formation of HCMV viral envelope. Transmission electron micrographs of recombinant HCMV.mVIP-infected HFtelo cells stably expressing the indicated shRNAs. Monolayers were infected with recombinant HCMV.mVIP at an moi of 2 and processed for EM at 7 dpi. Dox (2 µg/ml) was added on day 0 and 3. Multiple frames from each sample were imaged and photographed. Particles from a representative cell are shown. White and black arrows indicate non-enveloped particles and enveloped particles, respectively. Cyto, cytoplasm. Scale bar, 1 µm. The numbers of enveloped and non-enveloped particles were counted in each frame and calculated as a ratio of enveloped particles to total particles in the cytoplasm of infected cells. The graphs indicate the mean percent of the enveloped particles per total 10–30 particles in each frame (from >10 cells of each sample) ± SEM. (TIF) [file ppat.1003497.s006.tif]
